# Supplementary material for: Hybrid Models and Biological Model Reduction with PyDSTool
Source: PLoS Comput Biol. 2012 Aug 9;8(8):e1002628. doi: 10.1371/journal.pcbi.1002628 (PMC3415397; doi:10.1371/journal.pcbi.1002628)
Supplement: Text S4 — Complete source code for the PyDSTool package (version 0.88.120504). Includes API documentation and help files linking to web pages. This file is identical to the current public release on Sourceforge.net. (ZIP) [file pcbi.1002628.s004.zip › PyDSTool/html/PyDSTool.MProject.ModelTransform-class.html]

xml version="1.0" encoding="ascii"?


PyDSTool.MProject.ModelTransform


| Home | Trees | Indices | Help | PyDSTool |
| --- | --- | --- | --- | --- |

Package PyDSTool ::
Module MProject ::
Class ModelTransform

[hide private]

[frames] | no frames]

# Class ModelTransform

source code

```
object --+
         |
        ModelTransform
```

---

Model Transformer class.


|  |  |
| --- | --- |
| Instance Methods | [hide private] |

|  |  |
| --- | --- |
| \_\_init\_\_(self, name, model)  x.\_\_init\_\_(...) initializes x; see x.\_\_class\_\_.\_\_doc\_\_ for signature | source code |

|  |  |
| --- | --- |
| remove(self, obj)  Remove hybrid model generator | source code |

|  |  |
| --- | --- |
| add(self, obj)  Add hybrid model generator | source code |

|  |  |
| --- | --- |
| open\_gentrans(self, name)  Open a generator transformation transaction | source code |

|  |  |
| --- | --- |
| rollback\_gentrans(self) | source code |

|  |  |
| --- | --- |
| commit\_gentrans(self, new\_name, description=`'``'`) | source code |

|  |  |
| --- | --- |
| unresolved(self)  Returns the unresolved inconsistencies in model's internal interfaces. | source code |

|  |  |
| --- | --- |
| commit(self, new\_name)  Verifies internal interface consistency before returning new model spec. | source code |

**Inherited from `object`**:
`__delattr__`,
`__getattribute__`,
`__hash__`,
`__new__`,
`__reduce__`,
`__reduce_ex__`,
`__repr__`,
`__setattr__`,
`__str__`


|  |  |
| --- | --- |
| Properties | [hide private] |

**Inherited from `object`**:
`__class__`


|  |  |
| --- | --- |
| Method Details | [hide private] |

|  |  |
| --- | --- |
| \_\_init\_\_(self, name, model)  *(Constructor)* | source code |

x.\_\_init\_\_(...) initializes x; see x.\_\_class\_\_.\_\_doc\_\_ for
signature

Overrides: object.\_\_init\_\_: *(inherited documentation)*

  


| Home | Trees | Indices | Help | PyDSTool |
| --- | --- | --- | --- | --- |

|  |  |
| --- | --- |
| Generated by Epydoc 3.0.1 on Fri May 4 15:24:06 2012 | http://epydoc.sourceforge.net |
